# PIPET: predicting relevant subpopulations in single-cell data using phenotypic information from bulk data

**DOI:** 10.1093/bib/bbae260

**Published:** 2024-05-31

**Authors:** Xinjia Ruan, Yu Cheng, Yuqing Ye, Yuhang Wang, Xinyi Chen, Yuqing Yang, Tiantian Liu, Fangrong Yan

**Affiliations:** Research Center of Biostatistics and Computational Pharmacy, China Pharmaceutical University, Nanjing 211198, P.R. China; Research Center of Biostatistics and Computational Pharmacy, China Pharmaceutical University, Nanjing 211198, P.R. China; Research Center of Biostatistics and Computational Pharmacy, China Pharmaceutical University, Nanjing 211198, P.R. China; Research Center of Biostatistics and Computational Pharmacy, China Pharmaceutical University, Nanjing 211198, P.R. China; Research Center of Biostatistics and Computational Pharmacy, China Pharmaceutical University, Nanjing 211198, P.R. China; Research Center of Biostatistics and Computational Pharmacy, China Pharmaceutical University, Nanjing 211198, P.R. China; Research Center of Biostatistics and Computational Pharmacy, China Pharmaceutical University, Nanjing 211198, P.R. China; Research Center of Biostatistics and Computational Pharmacy, China Pharmaceutical University, Nanjing 211198, P.R. China

**Keywords:** single-cell, multiclassification, multi-omics, integration

## Abstract

Single-cell RNA sequencing has revealed cellular heterogeneity in complex tissues, notably benefiting research on diseases such as cancer. However, the integration of single-cell data from small samples with extensive clinical features in bulk data remains underexplored. In this study, we introduce PIPET, an algorithmic method for predicting relevant subpopulations in single-cell data based on multivariate phenotypic information from bulk data. PIPET generates feature vectors for each phenotype from differentially expressed genes in bulk data and then identifies relevant cellular subpopulations by assessing the similarity between single-cell data and these vectors. Subsequently, phenotype-related cell states can be analyzed based on these subpopulations. In simulated datasets, PIPET showed robust performance in predicting multiclassification cellular subpopulations. Application of PIPET to lung adenocarcinoma single-cell RNA sequencing data revealed cellular subpopulations with poor survival and associations with TP53 mutations. Similarly, in breast cancer single-cell data, PIPET identified cellular subpopulations associated with the PAM50 clinical subtypes and triple-negative breast cancer subtypes. Overall, PIPET effectively identified relevant cellular subpopulations in single-cell data, guided by phenotypic information from bulk data. This approach comprehensively delineates the molecular characteristics of each cellular subpopulation, offering insights into disease-related subpopulations and guiding personalized treatment strategies.

## Introduction

Bulk RNA sequencing (RNA-seq) combines numerous cells for sequencing, yielding the average gene expression level of the cell population. This approach provides an overview of gene expression but fails to delineate individual cell differences [1]. Single-cell sequencing technology has bridged this crucial research gap, with single-cell RNA-sequencing (scRNA-seq) solving the cell heterogeneity problem, which had not been solved through conventional transcriptome sequencing methods. This technology has contributed to advancing medical and biological research [2]. For example, single-cell studies have revealed genetic expression at the cellular level, enabling cell type identification, detection of tumor heterogeneity, differentiation of cell states, determination of resistant cell populations and characterization of complex cell populations, such as peripheral blood immune cells [3–5]. Despite over a decade of progress in single-cell sequencing, the high cost continues to limit sample sizes. It is challenging for us to conduct statistically significant analysis of clinical characteristics, such as prognosis, disease stages, drug resistance and treatment responses.

Large-scale RNA research has also matured, yielding rich clinical information accessible in public databases, including The Cancer Genome Atlas (TCGA) [6], Genomics of Drug Sensitivity in Cancer (GDSC) [7] and Gene Expression Omnibus (GEO) [8]. Consequently, correlating these clinical features with specific cellular subpopulations in single-cell data becomes imperative. On the one hand, few bioinformatics tools are available for integrating the clinical features of bulk data to identify relevant subpopulations in single-cell data. On the other hand, existing computational methods for integrating bulk data and single-cell data to identify phenotype-related subpopulations still have limitations in identifying multiclass phenotype-related cell subpopulations or in terms of generalizability [9–12]. Among the available tools, the widely used Scissor [11] algorithm quantifies similarity between each single-cell and bulk sample to discern cell subpopulations associated with specific phenotypes, albeit limited to binary prediction for categorical clinical features. Additionally, when classifying predictions, there tends to be a bias toward favoring ‘more similar’ categories, with negative correlation results typically lower than positive correlation results. Another algorithm, scAB [12], links single-cell data from scRNA-seq and scATAC-seq with bulk data through pairwise correlations, inferring cell states related to patient phenotypes through a matrix factorization model; however, scAB is applicable to survival and binary classification variables but cannot handle multivariate clinical characteristics.

To address the current tools’ limitations, we propose PIPET (Phenotypic information based on bulk data predicts relevant subpopulations in single-cell data), a versatile algorithm applicable to cross-platform data and multivariate predictions. Given an abundance of single-cell data, in designing PIPET, we considered data splitting and computational efficiency during parallel operations. Conversely, we also considered sparsity in single-cell data. PIPET offers an efficient method for predicting clinical phenotype-relevant subpopulations in single-cell data. Evaluation of multiple simulated datasets and validation using real tumor single-cell datasets with matching bulk clinical data demonstrated PIPET’s ability to infer cell subpopulations corresponding to multiclassification clinical phenotypes. This approach offers novel perspectives for analyzing single-cell subpopulations and provides insights into disease mechanisms and molecular characteristics.

## Methods

### Subpopulation identification in single-cell data using PIPET

(1) Establishing feature vectors for each subclass based on differentially expressed genes from bulk data

Regarding bulk data, we assumed a multiclass phenotype comprising $k$ categories ($k\ge 2$), with ${m}_A$ marker genes (differentially expressed genes filtered based on condition) in subclass $A$, ${m}_B$ marker genes in subclass $B$, … and ${m}_K$ marker genes in subclass $K$. We then constructed feature vectors based on the feature genes of each subclass. As shown in Supplementary Figure 1, for binary phenotype data ($k=2$)_,_ feature vectors ${F}_A$ of subclass $A$ and ${F}_B$ of subclass $B$ were composed of ${m}_A$ and ${m}_B$ marker genes, respectively. Considering marker genes of the two subclasses exhibited a positive and negative relationship, values of 1 were assigned to ${m}_A$ genes and −1 to ${m}_B$ genes in ${F}_A$_,_ with the same assignments used for ${F}_B$_._ For multiclass phenotype data ($k>2$), each subclass’ feature vector was composed of marker genes from all subclasses. To eliminate the influence of other subclasses for feature vector ${F}_K$ of subclass $K$, ${m}_K$ genes in subclass $K$ were assigned a value of 1, whereas marker genes from other subclasses were assigned a value of 0. Such assignment could facilitate the exploration of similarity between single-cell data and the feature vector of each subclass.

(2) Single-cell data preprocessing and final feature vector determination

Sparsity is a common challenge encountered in single-cell data analysis, where not all genes exhibit observable expression. To address this, filtering out genes with low expression frequency during preprocessing is crucial for enhancing data quality. This reduced the impact of uninformative long-tail data. Notably, in our PIPET tool, users have the flexibility to adjust the gene expression frequency parameter according to their specific analysis needs. They have the option to customize the elimination of genes with too low expression frequency, or include all genes in the analysis. After filtering genes in scRNA-seq data, we assumed that a single-cell expression matrix ${S}_{n\times c}$($n\ge{m}_A+...+{m}_K$) containing $c$ cells and $n$ genes was obtained. Taking the intersection of $n$ genes in the single-cell matrix and ${m}_A+...+{m}_K$ genes in the subclass feature vector, we obtained $N$ common genes. The final feature vector, ${F}_{1\times N}$, used for subsequent computations, contains only $N$ genes.

(3) Evaluating the similarity between single-cell data and each feature vector

We extracted the expression levels of $N$ genes from single-cell data, thereby forming a new single-cell expression matrix ${S}_{N\times c}$. Subsequently, we evaluated the distance or correlation between ${S}_{N\times c}$ and the feature vectors of $k$ subclasses. PIPET calculates the Euclidean, Manhattan and Chebyshev distances between ${S}_i$ and ${F}_j$ in single-cell data, thereby generating the distance ${d}_{ij}$($i=1,2,...,c$; $j=1,...,k$). Simultaneously, the cosine similarity and Pearson and Spearman correlation coefficients, ${r}_{ij}$, are calculated between ${S}_i$ and ${F}_j$(as shown below). By default, cosine similarity is used as the measuring method, with details on other methods provided in the supplementary materials.


$$ {r}_{ij}=\frac{S_i{F}_j}{\left\Vert{S}_i\right\Vert \times \left\Vert{F}_j\right\Vert } $$

(4) Calculating prediction confidence

We generated the null distribution by randomly resampling ${m}_A+...+{m}_K$ genes from $n$ genes of the single-cell expression matrix ${S}_{n\times c}$ for $nPerm$ iterations (flexible, default value is 1000). Prediction confidence was indicated by nominal *P*-values, estimated based on the null distribution for ${d}_i$ or ${r}_i$. Additionally, to account for multiple testing, we adjusted *P*-values using the Benjamini–Hochberg procedure.

(5) Determining subclasses corresponding to single-cell data

For each cell in the single-cell expression matrix, if the variable ${d}_{ij}$ generated with subclass $j$ was the minimum value or the variable ${r}_{ij}$ generated with subclass $j$ was the maximum value, we assigned the predicted label of subclass $j$ as the phenotypic information for that cell. However, we preferably considered the significance of *P*-values or adjusted *P*-values. Specifically, a threshold of *P* < 0.05 or false discovery rate (FDR) < 0.05 was used to annotate subclasses with significant associations.

### Simulation setup

We generated simulated scRNA-seq datasets containing 5000 cells and 5000 expressed genes based on the splatPop model [13] from the R package splatter. Gene information for the simulation was provided using the mockVCF and mockGFF functions. Cells were grouped based on condition.prob parameter with grouping probabilities set as follows: for two phenotypes, 0.6 and 0.4; for three phenotypes, 0.4, 0.4 and 0.2 and for four phenotypes, 0.2, 0.4, 0.2 and 0.2. To control cell-group effects, we set the similarity.scale parameter to 5. Parameters related to the condition effect, namely eqtl.n and eqtl.condition.specific, were set to 0.3 and 0.5, respectively. Dropout values were introduced into single-cell data by setting the dropout.type parameter to ‘experiment’ and adjusting dropout proportions through the dropout.mid and dropout.shape parameters. Subsequently, 30% of these cells were selected based on different phenotypes for further testing, whereas the remaining 70% were used to simulate bulk data. Bulk datasets for different phenotypes were created using the R package SimBu [14], with each dataset containing 50 pseudobulk samples, each comprising 500 cells. The simulation scenario selected was ‘random’, resulting in a raw count matrix with 50 samples for each phenotype.

### Sample information

Lung adenocarcinoma and breast cancer samples were obtained from TCGA. Transcriptome raw count data for TCGA-LUAD and TCGA-BRCA cohorts were downloaded via the TCGAbiolinks [15] package, and the corresponding clinical data were obtained from the Xena Public Data Center (https://xena.ucsc.edu/public-hubs). Low expression genes were filtered out by retaining genes with counts per million >1 in at least 10% of the samples. Filtered messenger RNAs were then annotated using the GENCODE 27 file. After matching gene expression data from 522 LUAD patients with clinical data, including age, survival and TP53 mutations, 437 patients with lung adenocarcinoma were included in downstream analysis. Similarly, after matching gene expression data, survival, PAM50 subtype, pathologic stage and other clinical data from 1174 BRCA patients, 1068 patients with breast cancer were included in subsequent analysis. Basic clinical information for each patient is provided in Supplementary Table S1.

scRNA-seq data for lung adenocarcinoma and breast cancer were obtained from published studies. Based on the lung adenocarcinoma single-cell dataset GSE131907 [16], we selected 11 tumors and 11 distant normal lung tissue samples for analysis. Breast cancer scRNA-seq data were derived from GSE176078 [17], including 11 estrogen receptor–positive (ER+), 5 human epidermal growth factor receptor 2 positive (HER2+) and 10 triple-negative breast cancer (TNBC) samples. These cohorts’ details are summarized in Supplementary Table S2.

### scRNA-seq data analysis

Seurat V4.3.0 [18] was used for preprocessing and analyzing scRNA-seq data. Given that published annotated data were used, cells and genes were not filtered again. However, preprocessing of single-cell data was conducted before performing uniform manifold approximation and projection (UMAP) for dimensionality reduction and visualization. The expression matrix was normalized using the NormalizeData function with default parameters. Highly variable genes were identified using the FindVariableFeatures function and the ‘vst’ method. Subsequently, data scaling was executed using the ScaleData function, followed by principal component analysis. Clustering was performed using the FindNeighbors and FindClusters functions. Furthermore, the FindAllMarkers function with the default Wilcoxon rank sum test method was employed to identify differentially expressed genes in PIPET cell subpopulations.

### Analysis of differential gene expression in bulk data

We used the R package DESeq2 [19] to perform analysis of differential gene expression based on the raw count expression matrix from bulk data. In the real data, filtering criteria included an FDR threshold of <0.05 and an absolute value of log2 fold change (log2 FC) exceeding 2. For the simulation data, we selected differentially expressed genes with FDR < 0.05 and |log2FC| > 1.

### Pathway enrichment analysis

For pathway enrichment analysis, we used the clusterprofiler [20] R package to assess the gene sets of interest. *P*-values were corrected for multiple testing using the Benjamini–Hochberg method. Kyoto Encyclopedia of Genes and Genomes (KEGG) and hallmark gene sets were downloaded from the R package msigdbr [21].

### Consistency assessment of multiclass data

Cohen’s kappa statistic [22] is a measure of classification accuracy. We applied the kappa coefficient to evaluate the agreement between categorical variables, defined as follows:


$$ k=\frac{P_0-{P}_e}{1-{P}_e}, $$

where ${P}_0$ indicates the accuracy of prediction and ${P}_e$ is the hypothetical probability of chance consistency.

### Survival analysis

We constructed a multivariate Cox regression model based on genes significantly differentially expressed in the poor prognosis subgroup. This was achieved using the coxph and survfit functions in the R package survival. Subsequently, we calculated the risk score for each sample based on regression coefficients obtained from the model. Samples were then divided into high-risk and low-risk groups using the median risk score. Kaplan–Meier curves were plotted using the survminer package, and log-rank tests were performed to assess differences in overall survival between groups. Furthermore, common clinical risk factors, such as age, gender and pathological stages, were included in multivariate Cox survival analysis to validate the prognostic signature’s ability and sensitivity in independently assessing patient prognosis. Throughout the analysis, *P* < 0.05 was considered statistically significant.

## Results

### Overview of PIPET

**Figure 1:**
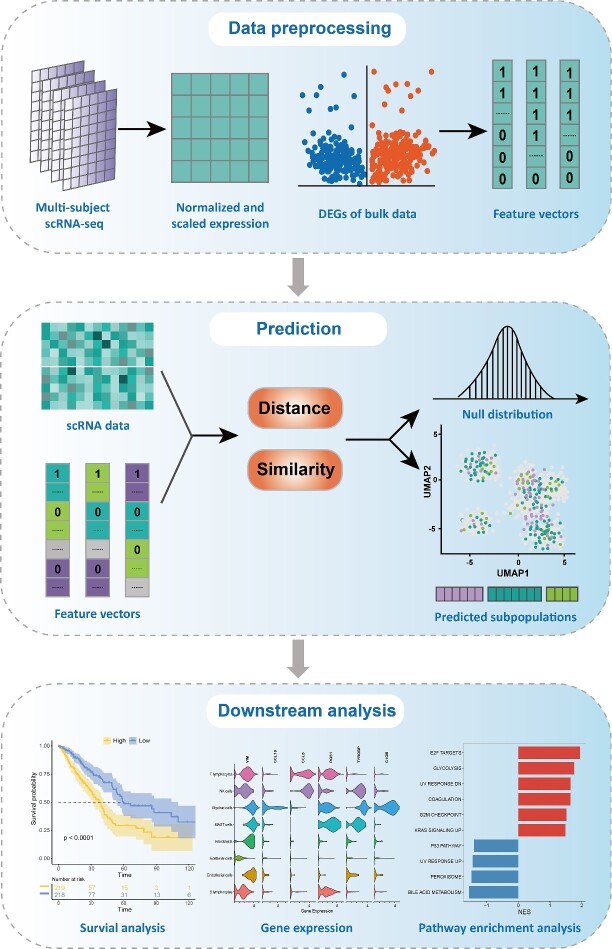
PIPET’s workflow, including data preparation, phenotype prediction and downstream analysis.

To leverage clinical information from bulk data to enrich single-cell data analysis, we developed PIPET (Figure 1), an algorithm for predicting relevant subpopulations in single-cell data based on the multiclass phenotypic information from bulk data. Initially, PIPET constructs feature vectors using the expression matrix and phenotype information of bulk data provided by the user or customizes feature vectors based on known differentially expressed genes. Following preprocessing of single-cell data, PIPET uses the single-cell expression matrix and corresponding feature vectors to predict phenotype-related subpopulations in single-cell data. The key step in PIPET involves evaluating similarity between single-cell data and each phenotype by calculating distance or correlation with feature vectors. Predicted confidence levels are considered to select highly correlated cell subpopulations with a given phenotype. After filtering based on *P*-values or FDR, the phenotype with the closest distance or highest correlation relative to the feature vector is labeled in the cell. Moreover, cells identified by PIPET can be further characterized in downstream analyses, e.g. visualizing cell distribution, exploring characteristic genes and analyzing pathway enrichment, with such analyses demonstrating PIPET’s ability to consistently identify cell subpopulations driving relevant phenotypes.

### Applying PIPET to simulated data

**Figure 2:**
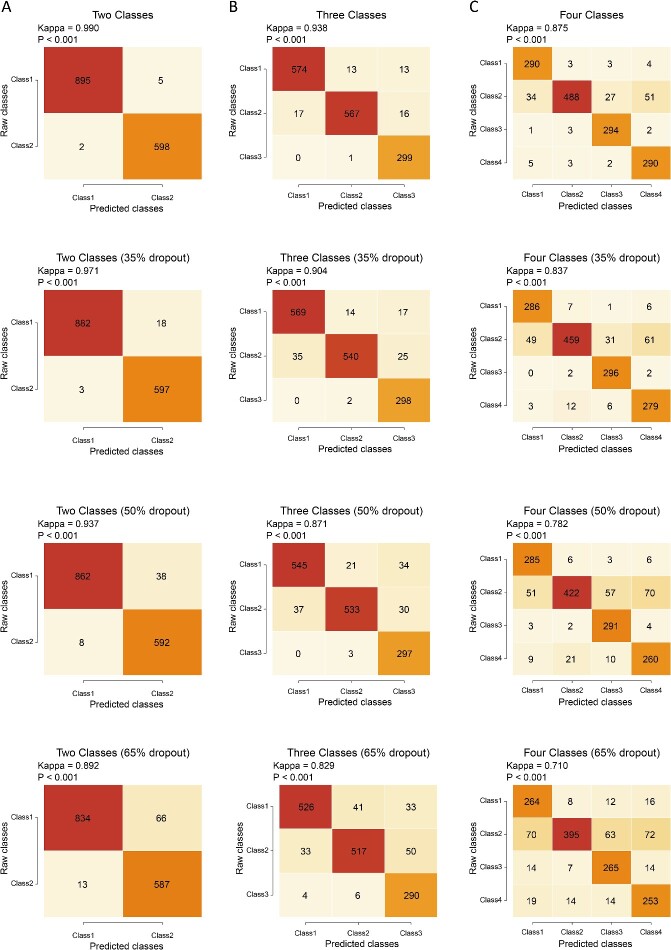
Consistency analysis results of (A) two-classification prediction, (B) three-classification prediction and (C) four-classification prediction with varying dropout proportions and without dropout.

Initially, we applied PIPET to multiple simulated datasets to assess its performance in predicting phenotypically relevant cell subpopulations. Using splatPop, scRNA-seq data with two, three and four phenotype-specific cell subpopulations were simulated. Subsequently, SimBu was employed to simulate pseudobulk RNA-seq data corresponding to each phenotype. In a simulation experiment with two phenotypes and no consideration of single-cell data dropout, 94 significantly differentially expressed genes were identified based on the bulk data, with 63 and 31 genes significantly upregulated in Class1 and Class2, respectively. PIPET’s predictions of relevant subpopulations in single-cell data aligned closely with the real phenotypic results, with a kappa statistic of 0.990 indicating high consistency. Additionally, we examined PIPET’s performance under different dropout proportions by generating simulated data with global dropouts. Under 35%, 50% and 65% dropout proportions, kappa values in consistency analysis were 0.971, 0.937 and 0.892, respectively (Figure 2A), indicating PIPET’s robustness to various dropout proportions. Extending the simulation and analysis process to data with three phenotypes and four phenotypes, PIPET demonstrated accurate identification of relevant cell subpopulations (Figure 2B and C), with kappa values of 0.938 and 0.875 for three and four phenotypes, respectively, when no dropouts were considered. Considering 35%, 50% and 65% dropout proportions, kappa values for three phenotypes were 0.904, 0.871 and 0.829, whereas those for four phenotypes were 0.837, 0.782 and 0.710, respectively. These results suggested that PIPET can accurately predict class in a multiclass scenario. In addition, we also conducted comprehensive simulation studies to assess the robustness of PIPET across various scenarios in the supplementary materials.

Comparing PIPET with the existing methods Scissor and scAB using simulated data with two phenotypes (as Scissor and scAB only support binary variables), the results revealed comparable performance in identifying phenotype-related cell subpopulations (refer to the supplementary materials).

In summary, we demonstrated the robustness of PIPET regarding sparse single-cell simulation data and multiclassification predictions. The predicted results were consistent with real phenotypes, indicating PIPET’s ability to identify cell subpopulations from single-cell data related to phenotypes in bulk data.

### Identifying characteristics of poor survival subpopulations in lung adenocarcinoma

**Figure 3:**
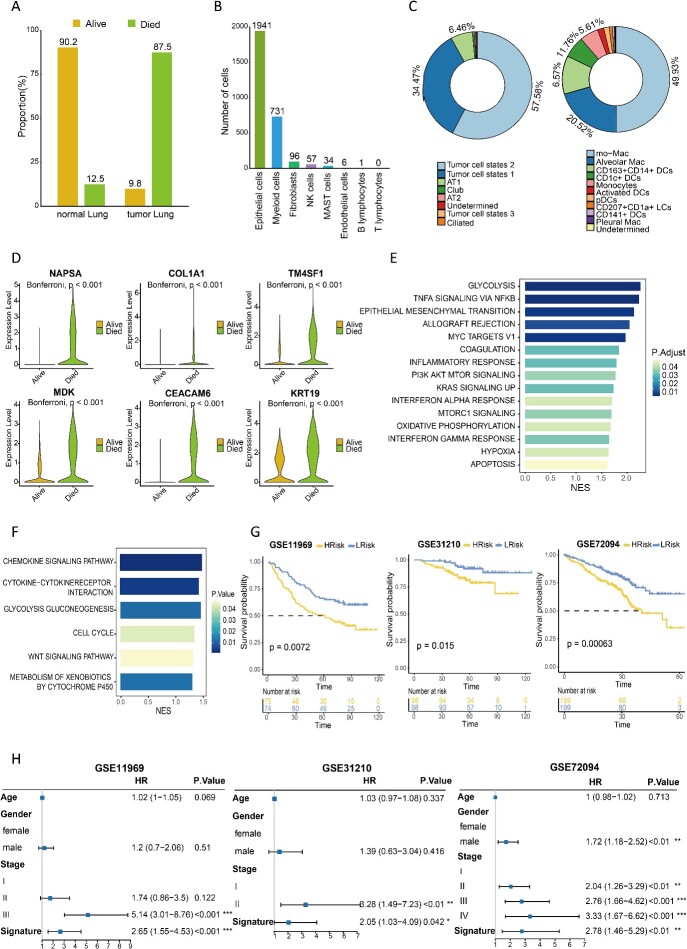
(A) Proportion of PIPET_Died and PIPET_Alive cells in normal and tumor tissue samples. (B) Distribution of each cell type in PIPET_Died cells. (C) Detailed distribution of epithelial cells and myeloid cells for PIPET_Died. (D) Expression levels of selected upregulated genes in PIPET_Died cells. (E) GSEA pathway analysis of the hallmark gene set. Adjusted *P*-values and normalized enrichment scores are depicted as bar colors and lengths, respectively. (F) KEGG pathway analysis of upregulated genes in PIPET_Died cells. (G) Kaplan–Meier curves of overall survival in low- or high-risk patients in three validation datasets. (H) Hazard ratios, 95% confidence intervals and *P*-values for the Cox model, including age, sex, pathological stages and our signature, in multivariate Cox survival analysis.

Based on survival information from 437 LUAD patients in TCGA, we employed PIPET to identify subpopulations associated with ‘alive’ and ‘died’ phenotypes in lung adenocarcinoma scRNA-seq data. After filtering by *P*-values, we identified 2866 cells significantly associated with poor survival (designated as PIPET_Died) and 814 cells significantly associated with good survival (designated as PIPET_Alive). Notably, 90.2% of PIPET_Alive cells were derived from single-cell samples of normal lung tissue, whereas 87.5% of PIPET_Died cells were from single-cell samples of tumor tissue (Figure 3A). Further analysis revealed that PIPET_Died cells were predominantly distributed among epithelial cells and myeloid cells (Figure 3B). For epithelial cells, two tumor cell states accounted for a substantial proportion of cells (tumor cell state 2: 57.58%; tumor cell state 1: 34.47%). In myeloid cells (Figure 3C), mo-Mac, associated with a marked impact on tumor invasiveness and metastasis, accounted for the largest proportion of cells (49.93%).

Comparison of PIPET_Died cells with PIPET_Alive cells revealed 12 significantly upregulated genes and 30 significantly downregulated genes in the former (adjusted *P* < 0.05, |log2 FC| > 2; Supplementary Table S3). Most upregulated genes were recognized prognostic markers for lung adenocarcinoma or associated with tumor progression and metastasis (Figure 3D). Pathway enrichment analysis showed that PIPET_Died cells were significantly enriched in hypoxia-related pathways (e.g. glycolysis and gluconeogenesis), inflammatory pathways (e.g. chemokine signaling and inflammatory responses) and pathways related to cell growth and development (e.g. the cell cycle and PI3K/AKT/mTOR signaling), among other related pathways (Figure 3E and F). We defined the 12 overexpressed genes in PIPET_Died as a signature associated with lung adenocarcinoma survival, constructing a risk score model. Application of this model to three independent lung adenocarcinoma datasets (GSE31210, GSE11969 and GSE72094; Supplementary Table S4) revealed worse prognosis for patients in the high-risk group compared with the low-risk group (Figure 3G). We also applied Scissor to these three validation cohorts; however, we observed poor performance in one of the validation cohorts (refer to the supplementary materials). Subsequent multivariable Cox survival analysis, including age, sex, pathological stages and our signature, demonstrated the notable impact of our signature on survival across the three external datasets (Figure 3H).

Collectively, these results emphasize PIPET’s ability to identify a subpopulation with poor survival from lung adenocarcinoma single-cell data. This subpopulation comprises more invasive cells and is associated with tumor progression and metastasis. Additionally, the identified signature of overexpressed genes holds potential as a prognostic marker for lung adenocarcinoma.

### Identification of cell subpopulations associated with TP53 mutations in lung adenocarcinoma

**Figure 4:**
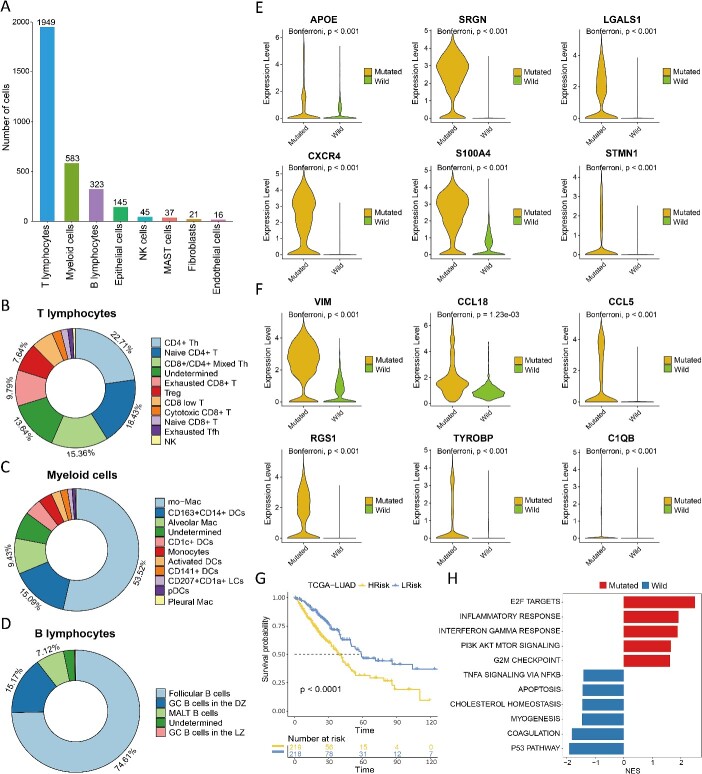
(A) Distribution of each cell type in PIPET_Mutated cells. (B–D) detailed distribution of T lymphocytes, myeloid cells and B lymphocytes for PIPET_Mutated cells. (E) Expression levels of selected upregulated genes contributing to cancer invasion and migration in PIPET_Mutated cells. (F) Expression levels of selected upregulated genes contributing to immunosuppressive environment formation and immune escape of tumor cells in PIPET_Mutated cells. (G) Kaplan–Meier curves of overall survival in low- or high-risk patients in the TCGA-LUAD data. (H) GSEA pathway analysis of the hallmark gene set. Normalized enrichment scores and cell subpopulations are depicted as bar length and different colors, respectively.

TP53 mutation is one of the most common mutations in patients with lung adenocarcinoma. We performed PIPET analysis based on 437 lung adenocarcinoma cases from TCGA, using TP53 mutation status (mutated or wild-type) as phenotypic information. Application of PIPET to tumor samples from lung adenocarcinoma scRNA-seq data revealed 3119 cells associated with mutant TP53 (designated PIPET_Mutated) and 2616 cells associated with wild-type TP53 (designated PIPET_Wild). Examination of PIPET_Mutated cells indicated their predominant distribution in three immune-related cell groups: T lymphocytes, myeloid cells and B lymphocytes (Figure 4A). T lymphocytes mainly included T helper, exhausted CD8+ T and Tregs cells (Figure 4B). Among B lymphocytes, follicular B cells and germinal center dark zone B cells constituted a large proportion of cells (Figure 4C). Among myeloid cells, mo − Mac and CD163 + CD14+ DCs were found in large proportions (Figure 4D), reflecting macrophage expansion and differentiation toward the anti-inflammatory phenotype M2 [23]. Combined with the high proportions of exhausted T and Treg cells, the PIPET_Mutated cell population represented an immunosuppressive profile.

Further analysis of gene expression differences between PIPET_Mutated and PIPET_Wild cells revealed significant differences, with 13 upregulated genes and 33 downregulated genes in PIPET_Mutated cells compared with PIPET_Wild cells (adjusted *P* < 0.05, |log2 FC| > 2; Supplementary Table S3). Some significantly upregulated genes were implicated in cancer invasion and migration (Figure 4E), potentially influencing tumor malignancy and prognosis, whereas others contributed to establishing an immunosuppressive environment and promoting the immune escape of tumor cells (Figure 4F). Combining these upregulated genes with survival information from TCGA lung adenocarcinoma cases confirmed that higher TP53 mutation signature scores were associated with a worse prognosis compared with lower signature scores (Figure 4G). Additionally, pathway enrichment analysis revealed activation of cell cycle–related pathways, such as E2F targets and G2M checkpoint, along with immune-related pathways, including inflammatory and interferon gamma responses, in PIPET_Mutated cells. Conversely, significantly enhanced pathways in PIPET_Wild cells included p53-related signaling and apoptosis pathways (Figure 4H).

Taken together, these findings emphasize the diverse immunosuppressive effects of TP53 mutations and their role in promoting immune escape of lung adenocarcinoma cells, potentially contributing to resistance mechanisms against checkpoint inhibitor therapy. In summary, our analysis reaffirms the accuracy of PIPET in identifying relevant cell subpopulations from single-cell data guided by phenotypic information.

### Detection of cell subpopulations associated with clinical subtypes in breast cancer

**Figure 5:**
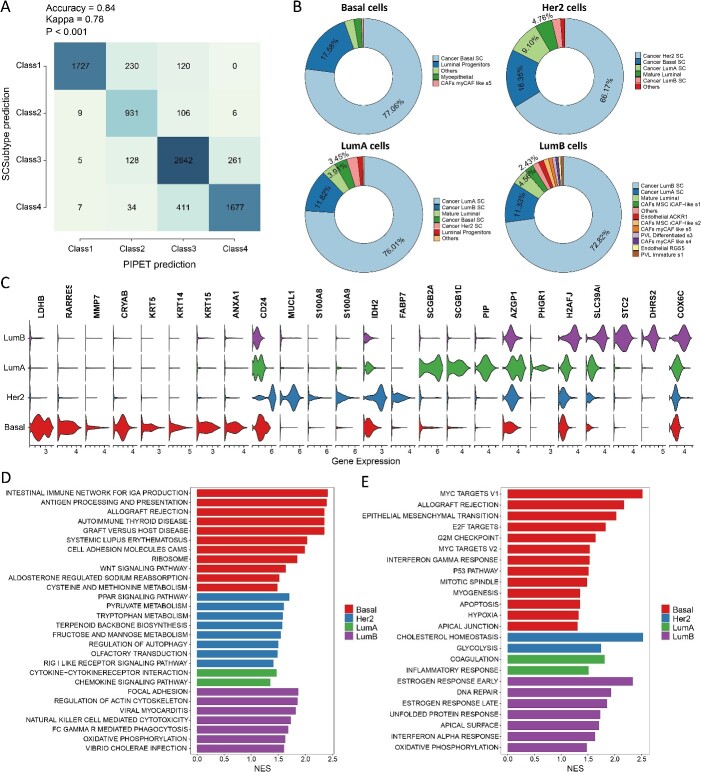
(A) Consistency analysis results between subtypes identified by PIPET and molecular subtypes defined by SCSubtype. (B) Distribution of PIPET_Basal, PIPET_Her2, PIPET_LumA and PIPET_LumB cells. (C) Expression levels of selected upregulated genes in PIPET_Basal, PIPET_Her2, PIPET_LumA and PIPET_LumB cell subpopulations. (D) KEGG pathway analysis. (E) GSEA pathway analysis of the hallmark gene set. In D and E, normalized enrichment scores and cell subpopulations are depicted by bar length and different colors, respectively.

The preceding analysis showed the efficacy of PIPET in binary predictions. To validate PIPET’s reliability in multiclassification predictions in single-cell data, we used PAM50 subtype signatures from TGGA-BRCA to guide the identification of cell subpopulations in breast cancer scRNA-seq data. PIPET identified 2241 cells related to the basal-like subtype (designated PIPET_Basal), 1407 cells related to the HER2-enriched subtype (designated PIPET_Her2), 3476 cells related to the luminal A subtype (designated PIPET_LumA) and 2303 cells related to the luminal B subtype (PIPET_LumB). PIPET’s identification results were consistent with the molecular subtypes defined by the original researcher using SCSubtype in breast cancer single-cell data, yielding a kappa value of 0.78 (Figure 5A). This suggests that basal-like breast cancers may originate from luminal progenitor cells, whereas HER2+ breast cancer may originate from both luminal progenitor cells and mature luminal cells, with luminal A and luminal B breast cancers originating from mature luminal cells [24]. Our findings generally align with this premise. Additionally, besides comprising subtype-related cells, PIPET_Basal cells are derived from luminal progenitor cells, whereas PIPET_Her2, PIPET_LumA and PIPET_LumB cells contain a small number of mature luminal cells (Figure 5B).

We also examined the genes characterizing each cell subpopulation identified by PIPET. Gene differential expression analysis revealed 43, 12, 9 and 37 significantly upregulated genes in PIPET_Basal, PIPET_Her2, PIPET_LumA and PIPET_LumB cell subpopulations, respectively (adjusted *P* < 0.05, |log2 FC| > 2; Supplementary Table S3). Violin plots displaying the expression of some upregulated genes are shown in Figure 5C. Notably, the genes *LDHB*, *MMP7*, *KRT5*, *KRT14* and *KRT15*, which were significantly upregulated in the PIPET_Basal subpopulation, are recognized as specific biomarkers of the basal-like molecular subtype or genes typically highly expressed in basal-like breast cancer. Furthermore, genes upregulated in PIPET_Her2 included multiple genes associated with HER2 positivity, such as *CD24*, *MUCL1* and *IDH2*. In the PIPET_LumA cell subpopulation, *AZGP1*, *PIP* and other genes known for their positive effect on patient prognosis were overexpressed. Conversely, genes highly expressed in PIPET_LumB, such as *STC2*, *COX6C* and *DHRS2*, may be associated with tumor invasion, metastasis and epithelial–mesenchymal transition in breast cancer. Based on KEGG pathway enrichment analysis (Figure 5D), pathways such as antigen processing and presentation, allograft rejection and Wnt signaling pathways were activated in PIPET_Basal. PIPET_Her2 exhibited enrichment in multiple metabolism-related pathways, whereas PIPET_LumA was enriched in inflammation-associated pathways, such as cytokine–cytokine receptor interaction and chemokine signaling. Notably, PIPET_LumB displayed enrichment in focal adhesion, natural killer cell–mediated cytotoxicity and oxidative phosphorylation, among other pathways. Hallmarks-based pathway enrichment analysis (Figure 5E) corroborated these findings, highlighting the significant upregulation of metabolism-related pathways in PIPET_Her2 and inflammation-related pathways in PIPET_LumA. Conversely, PIPET_Basal was characterized by pathways related to the cell cycle, cell proliferation and the epithelial–mesenchymal transition, whereas PIPET_LumB exhibited higher pathway activity in DNA repair and estrogen responses.

In summary, our PIPET analysis identified cell subpopulations in breast cancer single-cell data correlated with PAM50 subtypes. These subpopulations were closely aligned with those identified in previous studies, with each subpopulation exhibiting signature molecular characteristics.

### Analysis of cell subpopulations associated with TNBC subtypes

**Figure 6:**
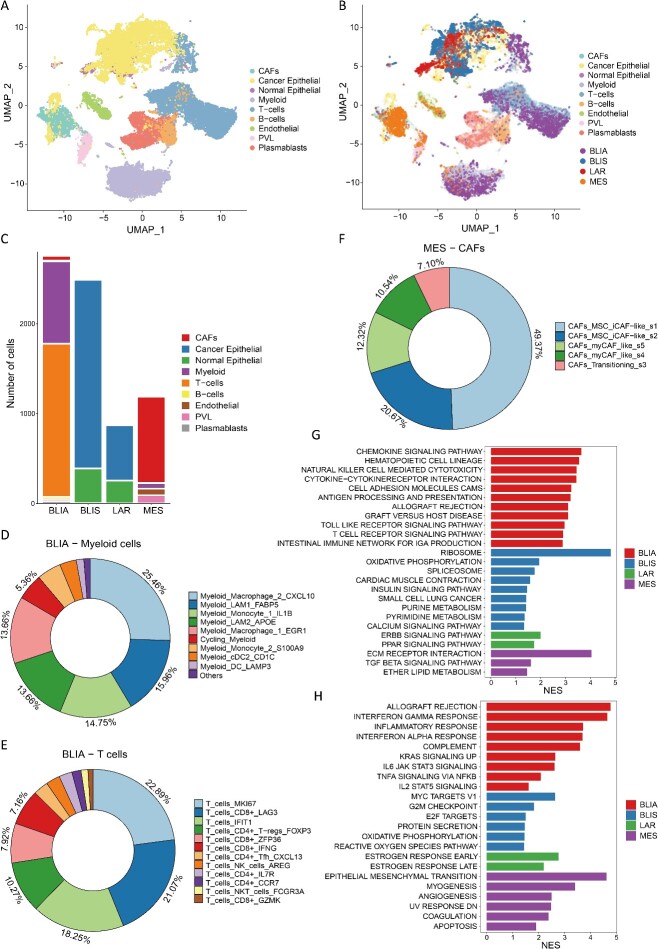
(A) UMAP visualization of TNBC cells with nine cell types. (B) UMAP visualization of PIPET_BLIA, PIPET_BLIS, PIPET_LAR and PIPET_MES cell subpopulations with TNBC cells as the background. (C) Distribution of each cell type in PIPET_BLIA, PIPET_BLIS, PIPET_LAR and PIPET_MES cells. (D) Detailed distribution of myeloid cells for PIPET_BLIA cells. (E) Detailed distribution of T cells for PIPET_BLIA cells. (F) Detailed distribution of CAFs for PIPET_MES cells. (G) KEGG pathway analysis. (H) GSEA pathway analysis of the hallmark gene set. In G and H, normalized enrichment scores and cell subpopulations are depicted by bar length and different colors, respectively.

To demonstrate the general applicability and flexibility of PIPET in exploring multiclass subpopulations in single-cell data, we used the TNBC subtype signature provided by Ding *et al*. [25] as phenotypic information to identify the related cell subpopulations in TNBC single-cell data. The signature is based on the classical TNBC subtypes defined by Burstein [26]: basal-like immune-activated (BLIA), basal-like immunosuppressed (BLIS), luminal androgen receptor (LAR) and mesenchymal (MES). Through PIPET, we generated 2753, 2489, 881 and 1189 cells related to the BLIA, BLIS, LAR and MES subtypes, respectively. The UMAP diagram shown in Figure 6 revealed that all TNBC cells belonged to nine cell types (Figure 6A), with the PIPET_BLIA, PIPET_BLIS, PIPET_LAR and PIPET_MES cell subpopulations derived from different cell types (Figure 6B). PIPET_BLIA was predominately derived from myeloid cells and T cells associated with immune regulation, whereas most PIPET_MES originated from cancer-associated fibroblasts (CAFs). Conversely, PIPET_BLIS and PIPET_LAR were primarily composed of cancer epithelial cells and normal epithelial cells (Figure 6C).

Further investigation of PIPET_BLIA cells revealed clusters related to the ‘M1-like’ phenotype (Mac_CXCL10), a cluster similar to the ‘M2-like’ phenotype (Mac_EGR1), a monocyte cluster with high IL1B expression (Mon_IL1B) and lipid-associated macrophage clusters (LAM1_FABP5 and LAM2_APOE) comprising high proportions in myeloid cells (Figure 6D). T cells (Figure 6E) mainly included a cluster associated with T cell proliferation (T_MKI67), a cluster driven by IFN-I features (T_IFIT1), a regulatory T cell cluster (CD4 + _Treg_FOXP3) and clusters including high expression of inhibitory checkpoint molecules (CD8 + _LAG3, CD8 + _IFNG and CD8 + _ZFP36). Whether representing clusters that enhance immune responses or those potentially involved in immunosuppression, PIPET_BLIA exhibited highly active immune regulation genes, distinguishing it from the other three cell subpopulations. Additionally, the PIPET_MES cell subpopulation mainly consisted of CAFs exhibiting characteristics of mesenchymal stem cells and inflammatory-like CAFs (Figure 6F), consistent with the definition of the MES subtype.

KEGG and hallmarks-based pathway enrichment analysis results (Figure 6G and H) revealed activated immune-related signaling pathways in the PIPET_BLIA cell subpopulation, including natural killer cell–mediated cytotoxicity, T cell receptor signaling, chemokine signaling, inflammatory responses, IL6/JAK/STAT3 signaling and IL2/STAT5 signaling. Additionally, the expression levels of *LYZ*, *C1QA*, *C1QB*, *C1QC*, *CCL4*, *CCL5* and *CXCL10/13*, among other genes, were significantly increased. The PIPET_BLIS subpopulation showed significant enrichment of cell cycle–related pathways, such as MYC targets, G2M checkpoint, E2F targets and certain metabolic pathways. *MUCL1* was overexpressed in the PIPET_LAR cells subpopulation, which also exhibited activated hormone-related pathways, including the estrogen response pathway and ERBB signaling pathway. In contrast, pathways related to tumor growth, invasion and metastasis (e.g. extracellular matrix receptor interaction, transforming growth factor-beta signaling pathway and the epithelial–mesenchymal transition) were significantly upregulated in the PIPET_MES cells subpopulation. These findings indicate that PIPET effectively identified cell subpopulations with characteristics consistent with the corresponding phenotype.

Through PIPET, we identified and analyzed four cell subpopulations related to TNBC subtypes, providing insights into the molecular mechanisms underlying these subtypes and potentially facilitating their diagnosis and treatment.

## Discussion

In recent years, scRNA-seq has gained traction for studying tumors, cardiovascular disease and other diseases [27]. However, its costly nature relative to bulk RNA-seq limits sample sizes [28]. Consequently, identifying phenotype-related cell subpopulations with statistical significance based on individual-level phenotypic information becomes challenging. Integrating extensive phenotypic information from bulk data with single-cell genomics data can address this limitation. Therefore, we introduced PIPET, a novel inference tool for predicting relevant cellular subpopulations in single-cell data based on the classification phenotype information from bulk data. Overall, this approach can provide insights into the clinical significance of single-cell data.

Despite the existence of computational methods for inferring cell subpopulations in single-cell data or integrating bulk and single-cell data to identify phenotype-related subpopulations, these methods remain limited in their ability to identify multiclass phenotype-related cell subpopulations. Some methods [29, 30] for inferring single-cell subpopulations are both highly targeted and lack versatility in terms of broader clinical information, such as disease stages, treatment responses and survival outcomes. Although Scissor [11] uses phenotypic information in bulk data to identify relevant cellular subpopulations, it is constrained by the types number of phenotypic information it can handle. Similarly, scAB [12] integrates single-cell genomics data with clinically annotated bulk data but faces limitations in handling multivariate categorical variables. In contrast, PIPET offers a user-friendly prediction method by integrating multivariate phenotypic information from bulk data to guide single-cell data analysis. It requires a single-cell expression matrix and a set of feature vectors containing phenotypic information as input to infer relevant cell subpopulations and return the prediction significance (*P*-value) for each cell. We used the nearest template prediction method [31], which has been successfully applied for subtype prediction in next-generation sequencing data, to integrate single-cell and bulk data and evaluate the similarity between single-cell data and each feature vector. By default, PIPET uses cosine similarity to evaluate similarity, although users can opt for other evaluation methods, such as Euclidean distance, Pearson correlation and Spearman correlation. PIPET’s flexibility extends to cross-platform and multiclassification predictions, eliminating the need for both unsupervised clustering of single-cell data and parameter optimization, thereby avoiding subjectivity in cell subpopulation classification and bias from parameter estimation.

We evaluated PIPET’s performance in predicting phenotype-associated cell subpopulations using simulated datasets with binary, triple and quadruple classification phenotypes. Despite a slight decrease in accuracy with increasing phenotype categories, PIPET showed robust prediction performance, even up to the four-category phenotype. Furthermore, we examined the impact of dropout values on PIPET performance in single-cell data, finding that PIPET could still identify relevant cell subpopulations effectively for binary and triple classification phenotypes. Despite a more substantial decrease in the kappa value for the four-category case, it remained within an acceptable range, indicating PIPET’s effectiveness in multiclassification predictions, albeit with a slight reduction in accuracy with excessive dropout values.

We also highlighted PIPET’s broad applicability in real data across different tumors and clinical phenotypes. We identified subpopulations associated with poorer survival in lung adenocarcinoma, as well as those related to TP53 mutations. Additionally, we analyzed subpopulations associated with clinical subtypes in breast cancer, identifying those related to TNBC subtypes. These results underscore PIPET’s ability to accurately identify relevant cell subpopulations from single-cell data guided by phenotypic information, each exhibiting signature molecular characteristics.

We acknowledge that this study has limitations that require further investigation. First, PIPET accommodates binary and multivariate categorical variables but cannot include survival in the analysis. Moreover, for continuous variables, such as age, PIPET is limited to categorizing them for analysis. Second, although PIPET performs well with scRNA-seq data, its effectiveness with single-cell data from other modalities, such as scATAC-seq data and single-cell proteomics data, remains unverified.

## Conclusions

In conclusion, PIPET effectively integrates single-cell data with bulk phenotypic information, allowing exploration of tissue heterogeneity in diseases. Through validation using simulated and real datasets, we showed that PIPET achieved superior performance in multiclassification phenotype identification. We anticipate PIPET’s application for cellular subpopulation identification in various research areas, including prognosis, drug resistance and treatment responses, which will facilitate disease mechanism discovery and promote personalized treatment strategies. Moreover, we have developed an R package to streamline PIPET’s use and accessibility, enabling users to easily apply it to their own datasets via the provided vignette (https://ruan2ruan.github.io/PIPET.html).

**Key Points**

- We present PIPET, a novel computational tool to integrate bulk phenotypic information with single-cell genomics data for the precise identification of cell subpopulations associated with various disease phenotypes.
- PIPET was rigorously validated using simulated and real datasets, demonstrating its superior performance in multiclassification phenotype identification.
- We provided an R package for PIPET, making our algorithm readily accessible to the scientific community and enabling researchers to apply it to their own datasets.
- PIPET had the potential to advance our understanding of disease heterogeneity, identify novel therapeutic targets and improve patient stratification for precision medicine approaches.

## Supplementary Material

Supplementary_Materials_bbae260

Supplementary_Table_S1_bbae260

Supplementary_Table_S2_bbae260

Supplementary_Table_S3_bbae260

Supplementary_Table_S4_bbae260

## Data Availability

The bulk data underlying this article were accessed from TCGA (https://www.portal.gdc.cancer.gov/repository) and Xena Public Data Hubs (https://xena.ucsc.edu/public-hubs). Single-cell datasets are available in the GEO data repository (https://www.ncbi.nlm.nih.gov/geo). Datasets derived from sources in the public domain are described in the ‘Methods’ section. The open-source PIPET R package is available in the GitHub online repository: https://github.com/ruan2ruan/PIPET.

## Author Biographies

**Xinjia Ruan** is a PhD student at the China Pharmaceutical University, who focuses on bioinformatics and cancer genomics.

**Yu Cheng** is a PhD student at the China Pharmaceutical University, who focuses on bioinformatics.

**Yuqing Ye** is a PhD student at the China Pharmaceutical University, who focuses on cancer genomics.

**Yuhang Wang** is a PhD student at the China Pharmaceutical University, who focuses on machine learning.

**Xinyi Chen** is a PhD student at the China Pharmaceutical University, who focuses on biostatistics.

**Yuqing Yang** is a PhD student at the China Pharmaceutical University, who focuses on biostatistics.

**Tiantian Liu** is an assistant professor at China Pharmaceutical University, who focuses on bioinformatics and cancer genomics.

**Fangrong Yan** is a professor of Biostatistics at the China Pharmaceutical University, who focuses on biostatistics and bioinformatics.
