# Supplementary material for: PIPET: predicting relevant subpopulations in single-cell data using phenotypic information from bulk data
Source: Brief Bioinform. 2024 May 31;25(4):bbae260. doi: 10.1093/bib/bbae260 (PMC11141296; doi:10.1093/bib/bbae260)
Supplement: Supplementary_Materials_bbae260 [file supplementary_materials_bbae260.docx]

**Supplementary Materials:**

**PIPET: Phenotypic information based on bulk data predicts relevant subpopulations in single cell data**

**Xinjia Ruan^1†^, Yu Cheng^1†^, Yuqing Ye^1^, Yuhang Wang^1^, Xinyi Chen^1^, Yuqing Yang^1^, Tiantian Liu^1*^, Fangrong Yan^1*^**

^1^ Research Center of Biostatistics and Computational Pharmacy, China Pharmaceutical University, Nanjing 211198, P.R. China.

^†^ These authors have contributed equally to this work.

*** Correspondence:**

Fangrong Yan, Research Center of Biostatistics and Computational Pharmacy, China Pharmaceutical University, Nanjing, PR China, 211198. E-mail: f.r.yan@163.com

Tiantian Liu, Research Center of Biostatistics and Computational Pharmacy, China Pharmaceutical University, Nanjing, PR China, 211198. E-mail: 632716883@qq.com

**Supplementary Text**

1. **Similarity evaluation methods in PIPET**

Assuming a single-cell expression matrix, $S_{N\times c}$, we evaluated the distance or correlation between $S_{N\times c}$ and the feature vectors of $k$ subclasses. Additionally, we generated the distance $d_{ij}$($i=1,2,...,c$; $j=1,...,k$) and correlation $r_{ij}$ between $S_{i}$ and $F_{j}$. The following methods are available in PIPET:

Cosine similarity: $r_{ij}=\frac{S_{i}F_{j}}{\left\|S_{i}\right\|\times\left\|F_{j}\right\|}$

Euclidean distance: $d_{ij}=\sqrt{\sum_{x=1}^{N} (S_{ix}-F_{jx})^{2}}$

Manhattan distance: $d_{ij}=\sum_{x=1}^{N} \left|S_{ix}-F_{jx}\right|$

Chebyshev distance: $d_{ij}=\max_{x} (\left|S_{ix}-F_{jx}\right|),x=[1,2,...,N]$

Pearson correlation coefficient: $r_{ij}=\frac{\sum_{x=1}^{N} \left(S_{ix}-\bar{S}_{i}\right)\left(F_{jx}-\bar{F}_{j}\right)}{\sqrt{\sum_{x=1}^{N} \left(S_{ix}-\bar{S}_{i}\right)^{2}}\sqrt{\sum_{x=1}^{N} \left(F_{jx}-\bar{F}_{j}\right)^{2}}}$

Spearman correlation coefficient: $r_{ij}=1-\frac{6\sum_{x=1}^{N} d_{x}^{2}}{N\left(N^{2}-1\right)}$,

where $d_{x}=\text{R(}S_{ix}\text{) - R(}F_{jx}\text{)}$ is the rank difference between each pair of samples, $S_{i}$ and $F_{j}$.

1. **Robustness evaluations of PIPET**

In this part, we evaluated the robustness of PIPET from multiple perspectives. First, we supplemented scRNA-seq simulation data of cell subpopulations with five and six phenotypes. The data were presented with 0.4, 0.2, 0.2, 0.1, 0.1 and 0.3, 0.2, 0.2, 0.1, 0.1, 0.1 as grouping probabilities, and the remaining parameters were the same as the text of the manuscript. After screening differentially expressed genes with FDR<0.05 and |log2FC|>1, we compared the prediction performance of PIPET for each classification phenotype under different dropouts rate (Supplementary Figure 2). The results showed that the kappa values of consistency evaluation decreased with the increase of phenotypes and the increase of dropouts rate. But when we took the adjusted P value into account, the kappa values stabilized. Even in the case of multi-classification and high dropouts rate, the kappa values can still remain above 0.9. Afterwards, we adjusted the number of differentially expressed genes to observe its impact on PIPET prediction performance. As shown in Supplementary Figure 3, when less than 20 marker genes were selected, the prediction results were very unstable. For simulation data with a dropout rate lower than 50%, 150 marker genes can maintain better multi-classification prediction results with a kappa value greater than 0.8. Therefore, considering real situation, computational runtime and the potential increase in gene overlap associated with selecting more marker genes, we recommend setting the number of selected genes to around 150. Finally, we also studied the impact of differentially expressed genes obtained by different tools on the PIPET multi-classification prediction results (Supplementary Figure 4). Under the same gene screening conditions, the differentially expressed gene results of DESeq2, edgeR, and limma did not affect the prediction of PIPET.

1. **Method comparisons**

**Applying Scissor to simulated data**. Using the Scissor vignette (<https://sunduanchen.github.io/Scissor/vignettes/Scissor_Tutorial.html>), we analyzed scRNA-seq data using the same simulated data for two phenotypes employed in PIPET (without considering dropout) as input. When alpha was set to 0.001 (with default parameters used in all other cases), 100% of cells was selected for identification. Scissor identified 591 Scissor+ cells (related to phenotype 2) and 909 Scissor− cells (related to phenotype 1), achieving a Kappa value of 0.988 for consistency evaluation.

**Applying scAB to simulated data**. scAB was used for analysis of scRNA-seq data based on the vignette provided at https://github.com/jinworks/scAB/blob/main/tutorial/scAB_demo.html. We used the same simulated data for two phenotypes employed in PIPET (without considering dropout) as input. Default parameters were applied, and the “select_K” function aided in selecting k. scAB only identified 81 and 92 cells associated with phenotype 1 and 2, respectively, excluding other cells from scAB selection. Since the number of cells identified by scAB is too small, the results are not comparable to our PIPET.

**Applying Scissor to three lung adenocarcinoma validation cohorts.** Using survival information from 437 TCGA-LUAD cases, Scissor identified 1459 Scissor+ cells (Scissor_Died) and 1985 Scissor− cells (Scissor_Alive). A comparison of Scissor_Died cells with Scissor_Alive cells revealed 13 significantly upregulated genes in Scissor_Died (adjusted P < 0.05, |log2 Fold change| > 3). Having constructed a risk score model related to lung cancer survival using these genes, we applied it to three validation cohorts. In the GSE31210 and GSE72094 datasets, overall survival was significantly shorter in the high-risk group than in the low-risk group (P = 0.0019 and P < 0.0001, respectively); however, no significant difference in overall survival was observed between these groups in the GSE11969 validation set (P = 0.56).

**Supplementary Figures**


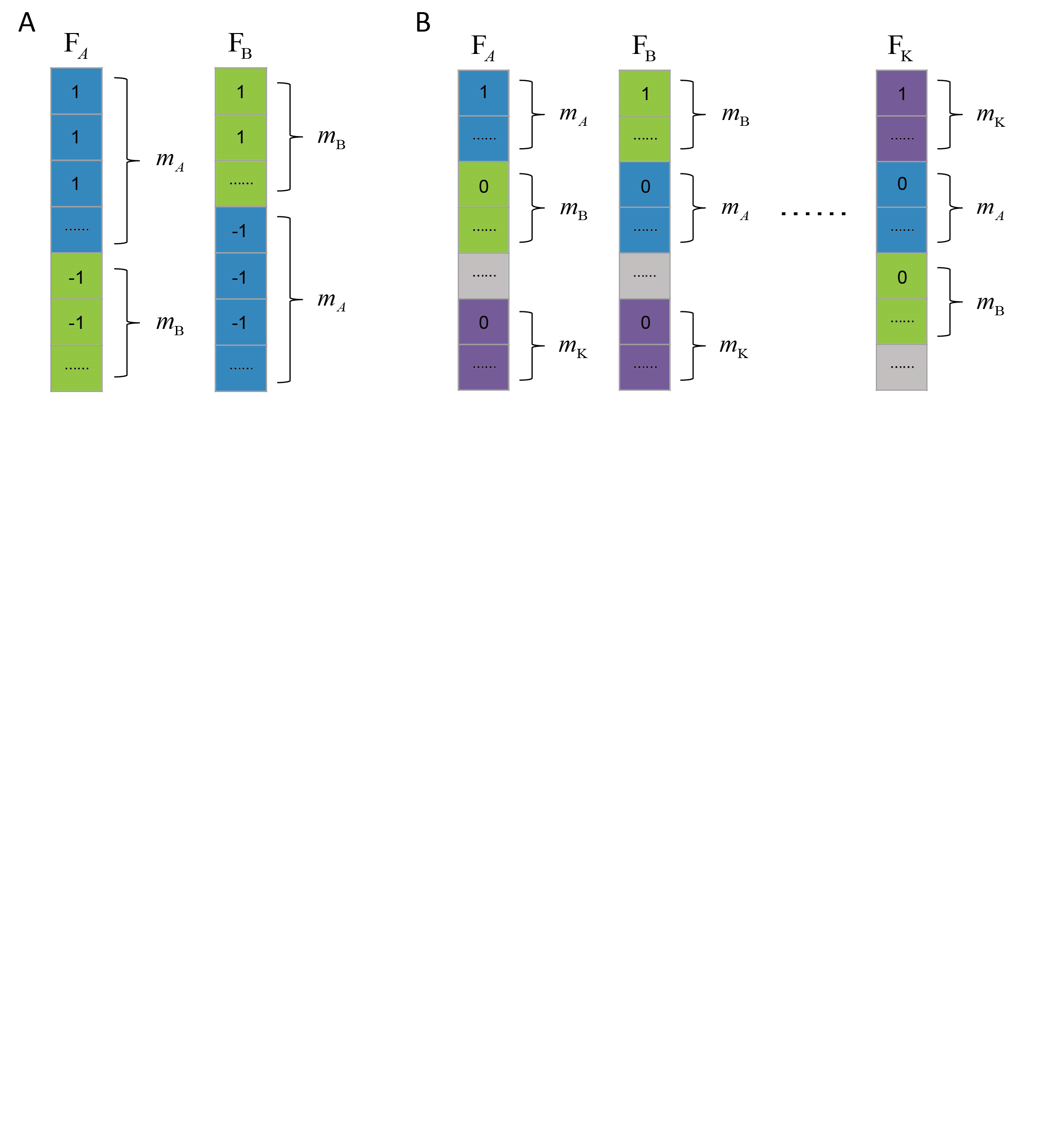


**Supplementary Figure 1. (A)** The composition of feature vectors corresponding to the binary phenotype data. **(B)** The composition of feature vectors corresponding to the multi-class phenotype data.


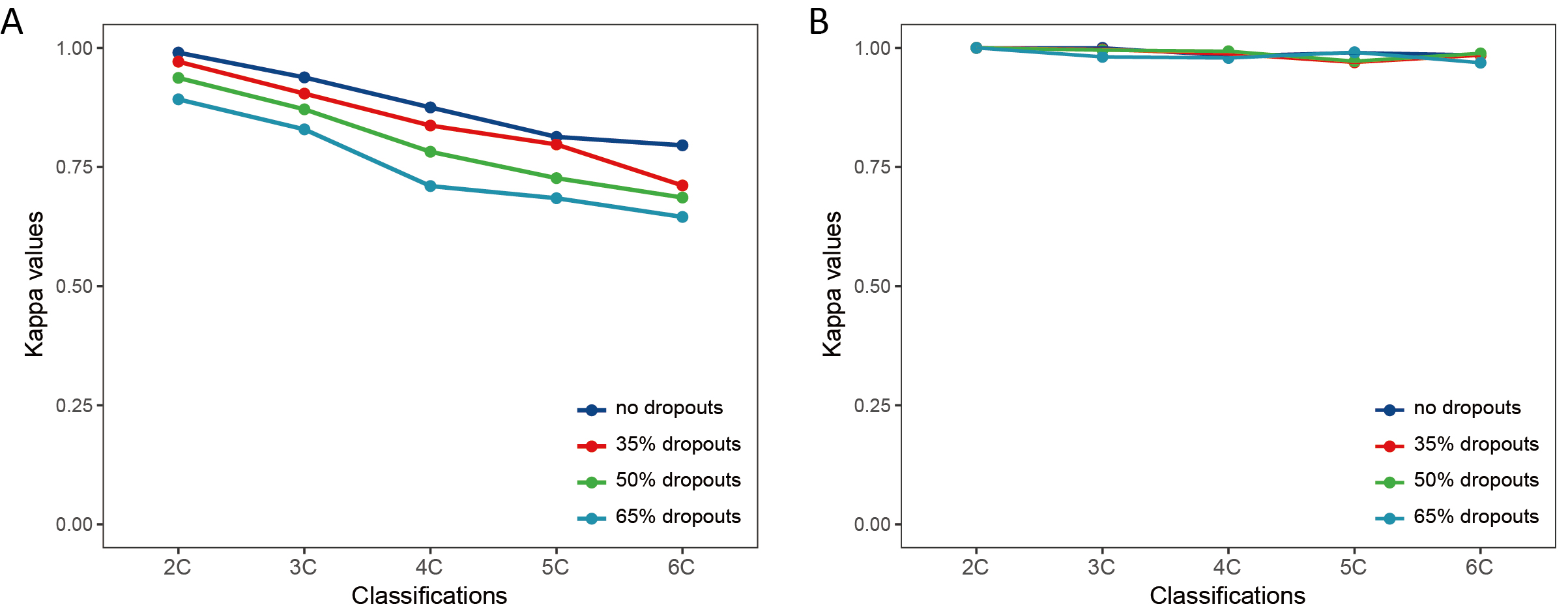


**Supplementary Figure 2.** (A) Consistency analysis results of different classification prediction by PIPET without adjusted P-values. (B) Consistency analysis results of different classification prediction by PIPET with adjusted P-values.


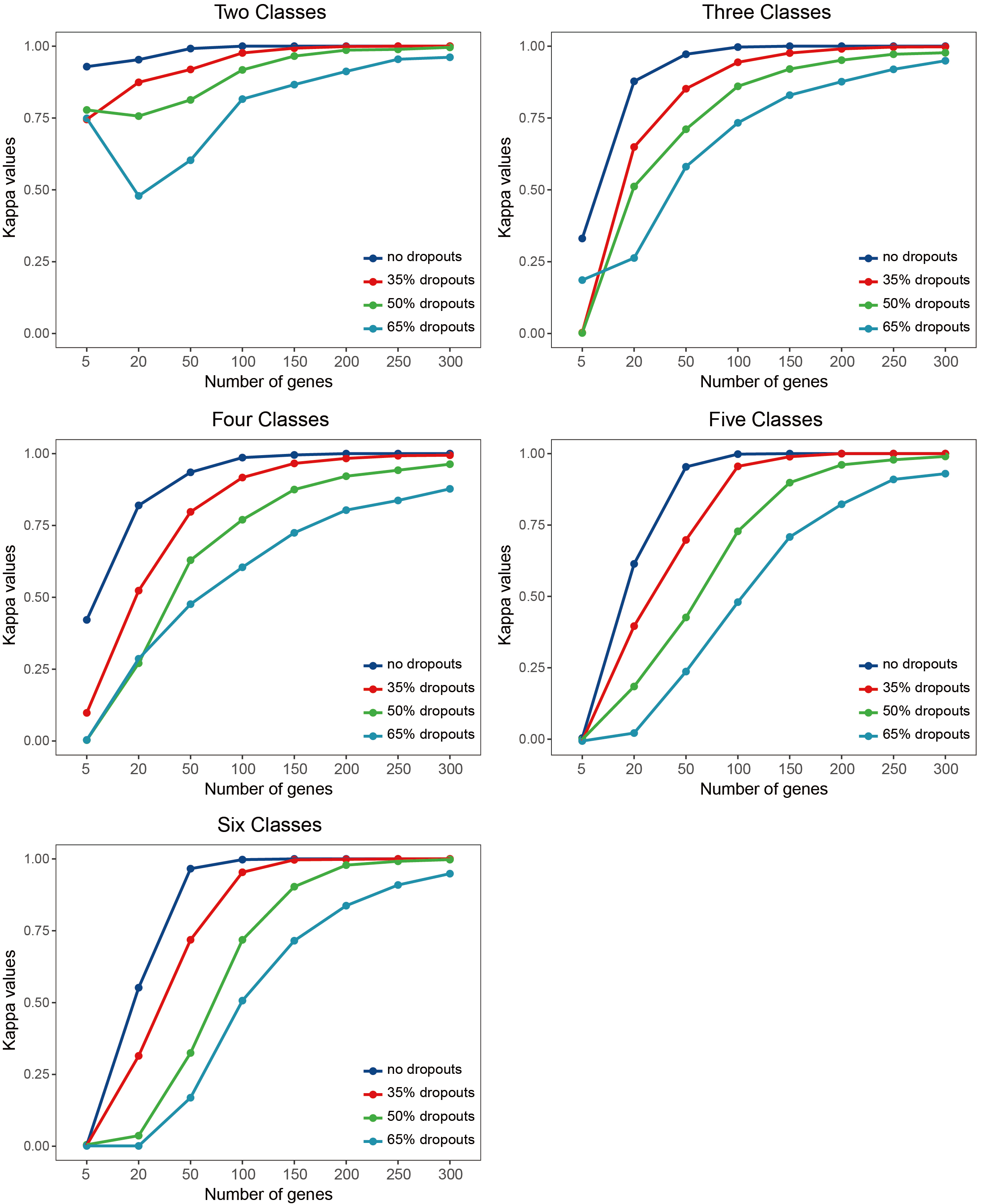


**Supplementary Figure 3.** Consistency analysis results of different classification prediction by PIPET with varying dropout proportions and without dropout.


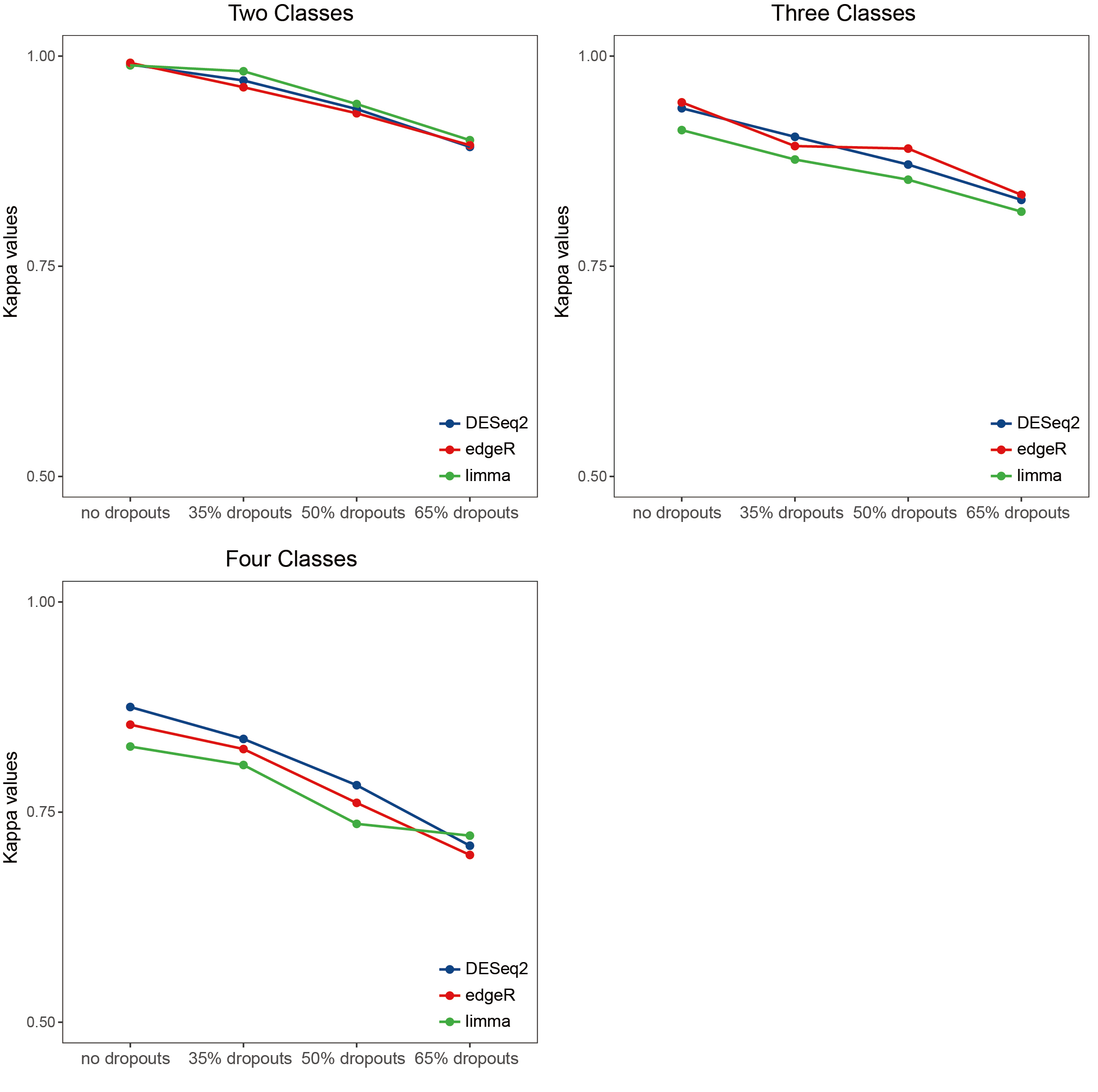


**Supplementary Figure 4.** Consistency analysis results of two, three, and four-classification prediction by PIPET using different software-selected DEGs with varying dropout proportions.

**Supplementary Tables**

**Supplementary Table S1.** Clinical information for TCGA-LUAD patients and TCGA-BRCA patients.

**Supplementary Table S2.** Basic information of single cell datasets.

**Supplementary Table S3.** Significantly expressed gene information in each cell subpopulations.

**Supplementary Table S4.** Basic information of validation datasets.
